# Case Report: A rare infection of multidrug-resistant *Aeromonas caviae* in a pediatric case with acute lymphoblastic leukemia and review of the literature

**DOI:** 10.3389/fped.2024.1233600

**Published:** 2024-05-13

**Authors:** Yiling Dai, Ju Gao, Mingyan Jiang

**Affiliations:** ^1^ Department of Pediatric Hematology and Oncology, West China Second University Hospital, Sichuan University, Chengdu, China; ^2^ Key Laboratory of Birth Defects and Related Diseases of Women and Children, Sichuan University, Ministry of Education, Chengdu, China

**Keywords:** *Aeromonas caviae*, bloodstream infection, pediatric acute lymphoblastic leukemia, drug sensitivity test, treatment

## Abstract

*Aeromonas caviae* infection of the bloodstream and intestine is a rare and severe opportunistic infection in immunocompromised people. In Southwest China, we first reported a case of bloodstream and intestinal infection with multidrug-resistant (MDR) *Aeromonas caviae* in a 4-year-old child with T-cell acute lymphoblastic leukemia. Blood and stool cultures were used to identify the infection. The selection of antibiotics was based on clinical expertise and medication sensitivity tests. We used linezolid, levofloxacin, and polymyxin B to treat the patient aggressively. *Aeromonas caviae* infection is uncommon in juvenile acute lymphoblastic leukemia. Doctors should be aware of the likelihood of opportunistic infection during the post-chemotherapy bone marrow suppression period. We further conducted a review of the literature and performed a detailed analysis of *Aeromona*s infection in pediatric leukemia. It is becoming increasingly apparent that antibiotic is abused domestically and abroad, resulting in the sharp increase of MDR bacteria. In general, most of the *Aeromonas* isolates are susceptible to third- or fourth-generation cephalosporins, aminoglycosides, quinolones, and carbapenem, but drug-resistant strains are being reported increasingly. We summarized the drug resistance rate of *Aeromona*s caviae and *Aeromonas hydrophila* in China in the last 10 years. Early recognition and effective treatment will improve prognosis and reduce mortality.

## Introduction

The *Aeromonas* genus is found ubiquitously in freshwater and soil environments. *Aeromonas* infection mainly occurs in patients with an underlying disease or immune deficiency and can cause serious multiple organ dysfunction (1). The incidence of *Aeromonas* septicemia was approximately 0.07 per 1,000 admissions, and in some regions, *Aeromonas hydrophila* and *A. caviae* are common pathogens (2, 3). The mortality rate, which can reach up to 24%–63%, not only increases the clinical work pressure but also threatens public health (4). Definitive diagnosis and appropriate antibiotic treatment are necessary to control disease progression.

Underlying hematological diseases, especially leukemia, apparently increase the likelihood and severity of illness caused by *Aeromonas*. Bloodstream infection is common among hematological diseases (5, 6). Over 90% of *Aeromonas* bloodstream infections occur during the neutropenic stage (7). Cytotoxic drugs and immunosuppressants can cause the reduction of neutrophils and a decrease in defense and immunity capacity. During this time, *Aeromonas* can invade the body through multiple pathways, especially damaged skin and mucosal.

In the past, *Aeromonas* strains were sensitive to third- or fourth-generation cephalosporins. Aminoglycosides, fluoroquinolones, and carbapenems are used empirically to treat multidrug-resistant (MDR) *Aeromonas* species. However, with the wide use of antibiotics in agriculture, fish farming, and clinical settings, increasing resistance has been noted (8, 9). The widespread application of broad-spectrum antibiotics has promoted the appearance of antimicrobial resistance genes (10, 11). An immunosuppressed individual is susceptible to opportunistic pathogen infection because of disease features. A retrospective study found that over 90% of *Aeromonas* bloodstream infections occur during the neutropenic stage. Leukemia patients with prolonged neutropenia are the high-risk population for *Aeromonas* infection (7, 12). The manifestations are not specific, but the infection often progresses rapidly. Hence, this case report aims to describe the clinical manifestation and summarize the treatment experience of MDR *A. caviae* infection in acute lymphoblastic leukemia.

## Case report

A 4-year-old girl admitted to West China Second University Hospital presented with epistaxis, ecchymosis, and paleness, followed by fever with a decreased neutrophil count (absolute neutrophil counts less than 1.5 × 10^9^/L). She was diagnosed with T-cell acute lymphoblastic leukemia with the *TLX1::TCRA* fusion gene after the morphology, immunology, cytogenetics, and molecular biology (MICM) classification. The brain, chest, and abdomen radiological examination showed a negative result, and no chromosomal abnormality or primary immune deficiency was detected. Before chemotherapy, cefoperazone/sulbactam (50 mg/kg q8 h) was given as an anti-infective regimen. She was stratified into the intermediate-risk group and successfully treated with induction therapy using the Chinese Children Cancer Group 2020 (CCCG-ALL-2020) protocol. The bone marrow response on day 19 (calculated from the start of chemotherapy) was evaluated using bone marrow aspiration and minimal residual disease (MRD). The bone marrow smear suggested complete remission, and the MRD was negative (less than 0.01%).

She developed significant neutropenia on day 4 of her first course of induction chemotherapy (measured from the commencement of chemotherapy). She also complained of mouth pain and face edema. Meropenem (40 mg/kg every 8 h) and vancomycin (15 mg/kg every 6 h) were administered as empiric antibiotics, and voriconazole (6 mg/kg every 12 h) was added as prophylactic for antifungal therapy because neutropenia persisted (for more than 14 days). Vancomycin and voriconazole blood concentrations were within normal limits. Her temperatures were consistently normal, and she had no additional signs of infection. On day 28, however, she developed a fever, diarrhea, and vomiting. Peritoneal discomfort and perianal skin erosion were discovered during a physical examination. Her neutrophil count was 0 × 10^9^/L, and her C-reactive protein (CRP) level was 110.1 mg/L. The ALL induction chemotherapy was not administered. Considering the risk of MDR Gram-positive coccus infection, vancomycin was changed to linezolid (10 mg/kg q8 h) and meropenem, and voriconazole was continuously administered at the same dose. However, just a day later (on day 29), she had septic shock, which manifested as wilt, hypoxemia, hypotension, and extended capillary refilling time. She was promptly transported to the Pediatric Intensive Care Unit after her blood volume was replenished. Noradrenaline and venoclysis hydrocortisone were administered, and she still had worsening dyspnea and continuous hypoxia.

**Figure 1:**
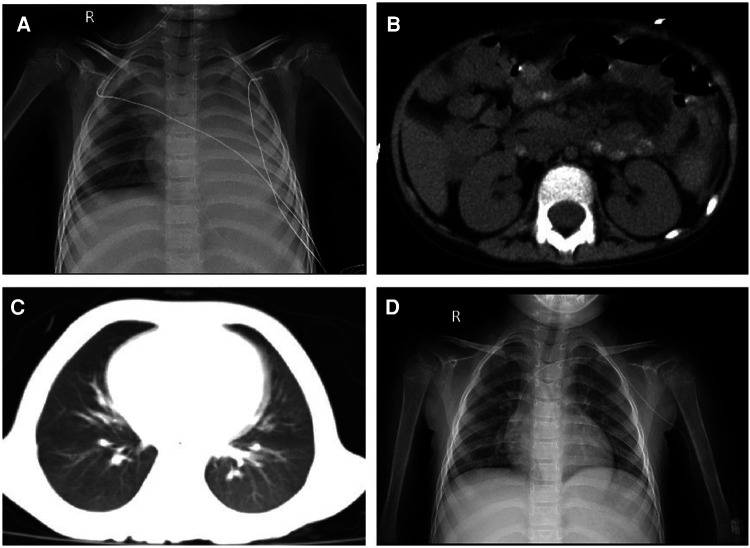
(**A**) Chest X-ray showing a significantly decreased transmittance in the left lung; (**B**) peritoneal thickening of the left paracolic sulcus; (**C**) chest CT showing a new exudative lesion in the post-basal segment of the left inferior pulmonary lobe; and (**D**) chest X-ray showing that the transmittance of the lung was improved.

The CRP and procalcitonin (PCT) increased to 299.8 mg/L and 23.67 ng/ml, respectively, with the disturbance of the internal environment, including hypoproteinemia, hyponatremia, hypokalemia, hypocalcemia, and hyperlactatemia (ALB 22.1 g/L, Na^+^ 128 mmol/L, K^+^ 2.82 mmol/L, Ca^2+^ 1.62 mmol/L, Lac 3.7 mmol/L). Aminotransferase, creatinine, and urea were normal. The stool culture identified MDR *A. caviae* (susceptible to amikacin, levofloxacin, and tigecycline) (Table 1). The microbial was cultured by Salmonella-Shigella agar medium and MacConkey agar medium. The microbial identification used matrix-assisted laser desorption ionization time-of-flight mass spectrometry (MALDI-TOF-MS) with 99% sensitivity. The micro-broth dilution method for antimicrobial susceptibility testing was used to determine the minimal inhibitive concentration (MIC) of antibiotics against the pathogen. The chest X-ray and abdomen computed tomography showed ground glass opacification in the post-basal segment of the left inferior pulmonary lobe and peritoneal thickening of the left paracolic sulcus (Figures 1A,B). Invasive mechanical ventilation was adopted, and antibiotics were adjusted to linezolid (10 mg/kg q8 h), amikacin (15 mg/kg qd), and tigecycline (1.2 mg/kg q12 h) simultaneously. Despite the shock subsiding through energetic treatment, she still had a high spiking fever, and the inflammatory cytokines did not descend to the normal level (CRP 183.7 mg/L, PCT 3.78 ng/ml). The 1,3-β-D glucan test and the serum galactomannan antigen (GM) test were negative. The cerebrospinal fluid examination was normal. However, the blood culture identified MDR *A. caviae* (susceptible to amikacin, levofloxacin, and tigecycline) and carbapenem-resistant *Acinetobacter baumannii* (susceptible to polymyxin B, tigecycline, aminoglycosides, and quinolones) (Table 1). The microbial was cultured using a Columbia blood agar base medium. Due to obstinate fever and persistent abnormal inflammatory cytokines, the antibiotics were adjusted to polymyxin B (1.5 million units/kg q12 h), levofloxacin (8 mg/kg q12 h), and linezolid (10 mg/kg q8 h) according to drug sensitivity results. On day 39, the temperature was back to normal, and the CRP and PCT decreased to 5.8 mg/L and 0.3 ng/ml, respectively. However, a repeat chest CT revealed a new exudative lesion in the post-basal segment of the left inferior pulmonary lobe (Figure 1C).

**Table 1:** Drug sensitivity test of *Aeromonas cavity* and *Acinetobacter baumannii.*

Samples	Bacterium	Antibiotics	Minimum inhibitory concentration, MIC	
			Quantitative	Qualitative
Stool	*A. cavity*	Cefoperazone/sulbactam	≥64.0	Resistant
	* *	Piperacillin/tazobactam	≥128.0	Resistant
	* *	Ceftazidime	16.0	Resistant
	* *	Cefepime	≥32.0	Resistant
	* *	Aztreonam	≥64.0	Resistant
	* *	Imipenem	≥16.0	Resistant
	* *	Meropenem	≥16.0	Resistant
	* *	Amikacin	≤2.0	Sensitive
	* *	Levofloxacin	1.0	Sensitive
	* *	Ciprofloxacin	2.0	Intermediate
	* *	Trimethoprim/sulfamethoxazole	≥320.0	Resistant
	* *	Tigecycline	≤0.5	Sensitive
Blood	*A. cavity*	Piperacillin/tazobactam	≥128.0	Resistant
		Cefoperazone/sulbactam	≥64.0	Resistant
		Ceftazidime	16.0	Resistant
		Cefepime	≥22.0	Resistant
		Aztreonam	≥64.0	Resistant
		Imipenem	≥16.0	Resistant
		Meropenem	≥16.0	Resistant
		Amikacin	≤2.0	Sensitive
		Levofloxacin	1.0	Sensitive
		Ciprofloxacin	2.0	Intermediate
		Trimethoprim/sulfamethoxazole	≥320.0	Resistant
		Tigecycline	≤0.5	Sensitive
Blood	*A. baumannii*	Piperacillin/tazobactam	≥128.0	Resistant
		Cefoperazone/sulbactam	≥64.0	Resistant
		Ticarcillin/clavulanat	≥128.0	Resistant
		Ceftazidime	≥64.0	Resistant
		Cefepime	≥32.0	Resistant
		Aztreonam	≥64.0	Resistant
		Meropenem	≥16.0	Resistant
		Imipenem	≥16.0	Resistant
		Amikacin	≤2.0	Sensitive
		Levofloxacin	0.5	Sensitive
		Ciprofloxacin	0.5	Sensitive
		Trimethoprim/sulfamethoxazole	160.0	Resistant
		Colistin	≤0.5	Sensitive
		Doxycycline	1.0	Sensitive
		Minocycline	≤1.0	Sensitive
		Tigecycline	1.0	Sensitive

**Figure 2:**
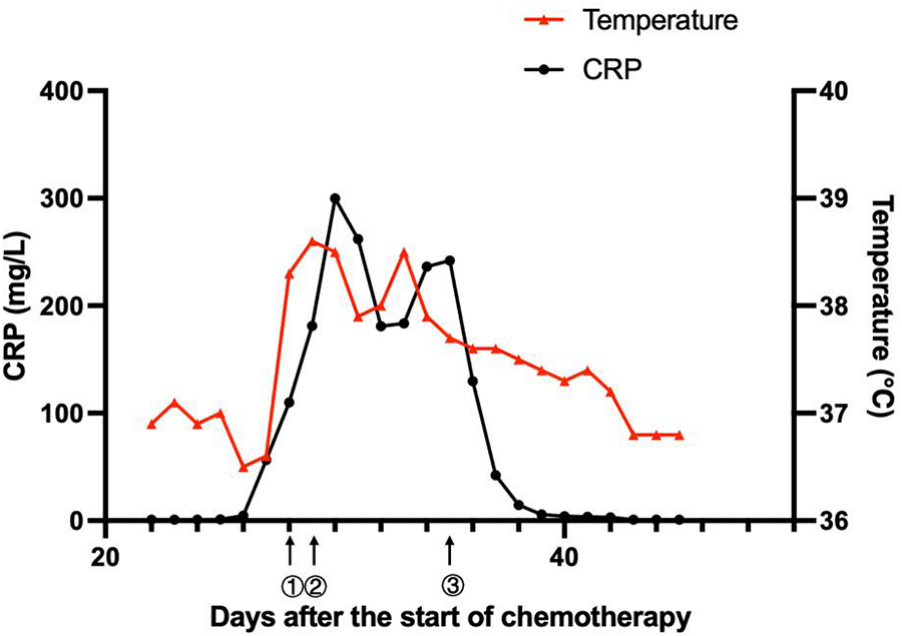
Clinical course of the patient. ① On day 28, the patient had a fever, diarrhea, and vomiting. The antibiotic therapy was switched to linezolid (10 mg/kg q8 h) combined with meropenem (40 mg/kg q8 h). ② On day 29, the patient suffered from septic shock. The antibiotics were adjusted to linezolid (10 mg/kg q8 h), amikacin (15 mg/kg qd), and tigecycline (1.2 mg/kg q12 h). ③ On day 35, with the child having an obstinate fever, persistent abnormal inflammatory cytokines, and drug susceptibility results, the antibiotics were adjusted to polymyxin B (1.5 million units/kg q12 h), levofloxacin (8 mg/kg q12 h) and linezolid (10 mg/kg q8 h).

On day 57, her neutrophils reverted to normal levels, and her pulmonary lesions in the chest X-ray showed no advancement (Figure 1D). She began the second round of induction therapy, which included cyclophosphamide, cytarabine, and mercaptopurine. The *A. caviae* and *A. baumannii* infections did not return after the treatment. Figure 2 depicts a chronology of the temperature and the C-reaction protein.

## Discussion

The *Aeromonas* genus is a bacterial group of cosmopolitan distribution and isolated from a broad range of sources like fresh water, soil, fruits, and vegetables (13). The first documented isolation of *Aeromonas* as a causative agent dates from 1954 in a Jamaican woman presenting with myositis (10). The bacteria are characterized facultatively as anaerobic, oxidase, and catalase-positive, Gram-negative bacillus and are considered opportunistic pathogens that mainly occur in patients with underlying diseases (14). The incidence of *Aeromonas* infection varies by geographical location and can be related to bad hygiene habits in underdeveloped regions (15). In pediatric patients, the *Aeromonas* genus is a common causal agent of intestinal infections, with *A. hydrophila* and *A. caviae* being the species mostly identified as causative agents (16). For the cases reviewed, we performed a detailed analysis of *Aeromonas* infection in pediatric leukemia (17–23) (Table 2). The seven cases are all younger than 18 years old, with a male-to-female ratio of 5:2, including acute lymphoblastic leukemia and acute myeloid leukemia. Only two subtypes have been found in the clinical samples, and the most predominant clinical species is *A. hydrophila*. The characteristics of the reported cases are summarized in Table 3. The common infection sites were the skin, soft tissue, and gastrointestinal tract. The clinical appearances were non-specific and included fever, pain, swelling, diarrhea, and vomiting. Before identifying the pathogen, penicillin and cephalosporin were used empirically. Once the diagnosis was clear, the antibiotics were adjusted to third- and fourth-generation cephalosporins, carbapenems, polymyxin, and amphotericin B. Three of the seven cases died due to *Aeromonas*-related sepsis (19, 20, 22).

**Table 2:** Different *Aeromonas* infections in pediatric leukemia.

Author	Age (years)	Gender	Nationality	Subtypes	Baseline Diagnosis	Prophylactic Antibiotics	Initiate Site	Infection Route	Symptoms	Proven Specimen	Treatment	Outcome
Papadakis et al. (17)	2.5	Female	Greece	*A. hydrophila*	B-ALL	Piperacillin/tazobactam Amikacin Fluconazole	Right thigh soft tissue	Bloodstream	Fever Hip pain Skin necrosis Gangrene	Blood culture	1. Daily tissue debridement 2. Granulocyte colony-stimulating factor 3. Cefepime (22 days) and tobramycin (20 days)	Favorable (articulatio coxae has full functionality)
Doganis et al. (18)	14	Female	Greece	*A. hydrophila*	AML-M0	Ceftazidime Gentamicin	Right lower limb soft tissue	Bloodstream	Fever Pain Swelling	Blood culture Pus culture	1. Teicoplanin (10 mg/kg/day, 2 weeks), liposomal amphotericin (3 mg/kg/day, 2 weeks), and ceftriaxone (100 mg/kg/day, 1 month) 2. Followed by 2 months of oral ciprofloxacin (15 mg/kg, q12 h) as well as IV γ-globulin monthly.	Death (the infection was under control, but 2 months later, the leukemia relapsed, and she died due to disease progress.)
Koçak et al. (19)	17	Female	Turkey	*A. sobria*	ALL	Imipenem	Digestive tract	Bloodstream	Abdominal pain Fever Hypoxemia Septic shock	Blood culture	Imipenem, linezolid, and ciprofloxacin	Death (due to *Aeromonas*-related sepsis)
Dean et al. (20)	16	Female	USA	*A. hydrophila*	AML	Penicillin Chloramphenicol	Right second metacarpal–phalangeal joint	Bloodstream	Fever Pain Swelling Erythema	Blood culture	Kanamycin and polymyxin B	Death (due to *Aeromonas*-related sepsis)
Shackelford et al. (21)	18	Male	USA	*A. hydrophila*	AML-M0 (relapse)	Penicillin	Right lower limb skin	Wound	Fever Vomiting Skin lesion Erythema	Pus culture	1. Erythromycin (75 mg/kg/day) and gentamicin (5 mg/kg/day) 2. Followed by oral chloramphenicol	Favorable
Moyer et al. (22)	4	Female	England	*A. hydrophila*	ALL	Penicillin	Digestive tract	Bloodstream	Fever Diarrhea Vomiting Abdominal pain Skin nodules	Blood culture Gangrenous skin lesions	Carbenicillin (600 mg/kg/day), probenecid (40 mg/kg/day, orally), Colomycin (50,000 units/kg/day), and gentamicin (3 mg/kg/day).	Death (due to *Aeromonas*-related sepsis)
Abrams et al. (23)	14	Male	USA	*A. hydrophila*	AML	Polymyxin B cephalothin	Digestive tract	Bloodstream	Fever Vomiting Abdominal distension Ileus	Blood culture	Chloramphenicol (1 g, q6 h) and kanamycin (150 mg, q6 h)	Death (due to systemic fungus infection)

**Table 3:** Characteristics of reported cases (17–23).

Characteristics	Reports (number)	Ratio (%)
Gender	** **	** **
Male	2	28.5
Female	5	71.5
Baseline diagnosis	** **	** **
ALL	3	42.8
AML	4	57.2
Subtypes	** **	** **
*A. sobria*	1	14.2
*A. hydrophila*	6	85.8
Infection site		
Skin	1	14.2
Soft tissue	3	42.9
Gastrointestinal tract	3	42.9
Infection route	** **	** **
Wound	1	14.2
Bloodstream	6	85.8

Although *Aeromonas* infection is usually reported in immunocompromised hosts, MDR *A. caviae* infection among children with leukemia is rare. Here, we first reported a case of bloodstream and intestinal infection by MDR *A. caviae* in pediatric acute lymphoblastic leukemia. Considering that *A. caviae* is an opportunistic pathogen, the risk factors of infection are indeed a matter of great concern to us. Through a thorough search for risk factors, it would be reasonable to suppose that the infection occurrence is related to the immunocompromised status induced by post-chemotherapeutic bone marrow suppression. In addition, the patients came from regions with a relatively backward economy and poor sanitation and nutritional status, which may increase the risk of severe infection. Improving the living environment and hygiene practices and strengthening nutritional support are beneficial for preventing the infection.

*Aeromonas* infection causes a wide range of clinical symptoms, from acute self-limiting diarrhea to systemic inflammatory response syndrome (SIRS) (24). Acute gastroenteritis is one of the most common manifestations among healthy people, and the major cause is the consumption of contaminated food (25). *Aeromonas*-related gastroenteritis most usually manifests as self-limiting watery diarrhea, but it can also manifest more severely as dysentery- and cholera-like disease (26). Primary or spontaneous *Aeromonas*-related peritonitis is a severe consequence in the elderly or immunocompromised population due to impaired intestinal motility and inadequate host defenses (27). *Aeromonas* can also cause a variety of skin and soft tissue infections, particularly in people who have been injured or have been exposed to a contaminated aquatic environment (28). The common *Aeromonas* skin infections are impetigo, erysipelas, cellulitis, folliculitis, abscesses, and necrotizing fasciitis, and the reported common subtype is *A. hydrophila* (24, 25). *A. caviae*-associated skin and soft tissue infection can be seen in some sporadic cases. One case involved a 22-year-old healthy Korean female, who underwent cosmetic liposuction and presented to the hospital with necrotizing fasciitis in both calves. She subsequently developed multiorgan dysfunction and skin necrosis with consequent massive skin loss. Culture reports on wound swabs and debrided tissue specimens proved an *A. caviae* infection. Through broad-spectrum antibiotics and repeated aggressive wound exploration and debridement, she eventually recovered from this severe invasive infection (26). *Aeromonas* septicemia has a fulminant course, with its mortality rate ranging from 25 to 30%. It is usually reported in immunocompromised hosts. The signs or symptoms do not distinguish from systemic infections caused by other bacteria; therefore, diagnosis needs to rely on pathogenic evidence (10, 27). An increasing number of laboratories and medical institutions have started to adopt next-generation sequencing (NGS) to search for rare pathogens. NGS is a rapid and non-invasive diagnostic method. Using NGS is recommended when an infection with a rare pathogen is suspected, especially immunocompromised individuals who need emergency treatment. *Aeromonas* meningitis is an uncommon clinical entity, and few cases have been described in pediatrics. The high risks are associated with hematological disease, surgical craniotomy, and traumatic skull fracture (28, 29). We recommend that intracranial examinations, including head computed tomography and cerebrospinal fluid examination, should be implemented as soon as possible, even if the patient does not have neurological signs and symptoms.

**Figure 3:**
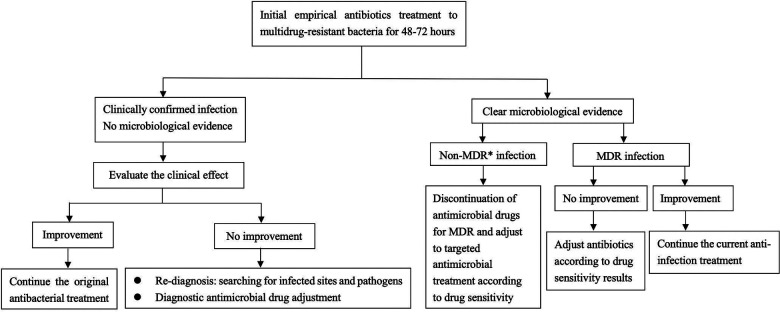
Diagnosis and treatment flow chart of multidrug-resistant bacteria. *MDR multidrug-resistant bacteria.

The patient we reported was in the period of post-chemotherapeutic bone marrow suppression. During the therapy, she developed persistent fever, diarrhea, vomiting, and perianal skin erosion. The clinical manifestations resembled infections with other pathogens, but she rapidly developed septic shock. Clinical manifestation and stool culture results highly indicated an enterogenic infection. The patient was hospitalized for chemotherapy, but the diet was prepared by her parents outside the hospital based on their wishes and demands. The intake of contaminated food or water was the possible infection route. Through blood and stool culture, *A. caviae* was eventually identified. The early symptoms of *A. caviae* infection are not typical, but the clinical course proceeded rapidly. The diagnosis relies on etiology, that is, once the *Aeromonas* are confirmed by sterile body fluid or secretion culture (blood, cerebrospinal fluid, sputum, urine, stool, and wound secretions), potent antibiotics are then immediately adopted. The use of antibiotics in clinical practice is undoubtedly a revolutionary milestone in medicine, and antibiotics are on the front line in the fight against *Aeromonas* infection. *Aeromonas* are resistant to penicillin and first-generation cephalosporins due to beta-lactamases, but they are susceptible to third- and fourth-generation cephalosporins, aminoglycosides, and fluoroquinolones. Our patient was also infected with carbapenem-resistant *A. baumannii*, which was equally worrying because the coexistence of two bacteria may exacerbate the patient's condition. We eventually chose levofloxacin and polymyxin B based on the drug sensitivity test of two bacteria. Her temperatures and levels of inflammatory cytokines were back to normal through regular potent antibiotic treatment. We further summarized herein the drug resistance rate of *A. caviae* and *A. hydrophila* in China for the last 10 years (Tables 4, 5). The *Aeromonas* strains extracted from the literature all corresponded to clinical cases. They were mainly isolated from the bile, followed by wound secretion and blood (30–35). The uncontrolled and indiscriminate usage of antibiotics has prompted the generation of drug-resistant bacteria. The spread of MDR bacteria in various situations has resulted in an inescapable public health issue: the number of effective antibiotics accessible for treatment is decreasing. Some hospital strains are resistant to numerous medicines, including carbapenems, colistin, and tigecycline, which are utilized as a last resort in situations involving multiply-resistant bacteria (34, 35). We summarized the MDR bacteria diagnosis and treatment flow chart in Figure 3, along with three clinical treatment experiences: first, early detection of MDR bacteria is critical; second, understanding the evolution of pathogen resistance profiles is critical for providing effective treatments; through the national or regional antimicrobial surveillance network, the types and proportions of pathogens can dynamically be investigated; the communication between laboratorians and clinicians should be further improved to strengthen hospital infection control and stewardship of antimicrobial agents; and third, therapeutic administration should consider the risk factors, clinical manifestations, and drug susceptibility and should not be limited to specific drugs.

**Table 4:** Drug resistance rate of *A. caviae* in China for the last 10 years (30–35).

Category	Medicine	Drug resistance rate (%)		
		2012	2018	2020
Penicillin	Ampicillin	100	93.55	100
	Piperacillin tazobactam	10	14.52	26.2
Cephalosporin	Cefazolin	93.3	95.16	90.6
	Ceftazidime	23.3	41.94	45.2
	Ceftriaxone	16.7	54.84	52.4
Aminoglycosides	Gentamicin	10	11.29	30.2
	Amikacin	0	3.23	4.7
Quinolones	Levofloxacin	6.45	11.29	26.2
Carbapenems	Imipenem	0	4.84	15.9

**Table 5:** Drug resistance rate of *A. hydrophila* in China for the last 10 years (30–35).

Category	Medicine	Drug resistance rate (%)				
		2010	2013	2016	2019	2020
Penicillin	Piperacillin tazobactam	9.5	2.7	3.2	0	10.26
Cephalosporin	Ceftazidime	15.0	20.6	16.1	13.64	16.67
	Ceftriaxone	15.0	28.6	32.3	8.33	7.1
Aminoglycosides	Gentamicin	5.0	8.8	6.7	0	7.69
	Amikacin	0	9.1	0	7.69	1.28
Quinolones	Levofloxacin	0	0	9.7	13.04	11.54
Carbapenems	Imipenem	0	8.3	3.2	0	3.85

## Conclusion

*Aeromonas* are common aquatic bacteria that are widely spread in the environment and can infect humans and animals. The presence of an underlying disease, particularly neoplastic disease, appears to enhance the risk of *Aeromonas* infection and the disease severity. Ever since the widespread use of antibiotics, drug-resistant microorganisms, particularly carbapenem-resistant bacteria, have proliferated. Under the management of antibiotic selection, early identification and reasonable antibiotic selection are crucial for improving prognosis and reducing mortality.

## Data Availability

The raw data supporting the conclusions of this article will be made available by the authors, without undue reservation.
